# Ketogenic diet for treating alopecia in BCS1l‐related mitochondrial disease (Bjornstad syndrome)

**DOI:** 10.1002/jmd2.12109

**Published:** 2020-03-25

**Authors:** Adela Della Marina, Baerbel Leiendecker, Sebastian Roesch, Saskia B. Wortmann

**Affiliations:** ^1^ Department of Neuropediatrics, Developmental Neurology and Social Pediatrics University of Duisburg‐Essen, Children's Hospital Essen Germany; ^2^ Department of Otorhinolaryngology, Head and Neck Surgery Paracelsus Medical University Salzburg Austria; ^3^ University Children's Hospital, Paracelsus Medical University Salzburg Austria; ^4^ Institute of Human Genetics Technical University München Munich Germany; ^5^ Institute of Human Genetics, Helmholtz Zentrum Neuherberg Germany; ^6^ Radboud Center for Mitochondrial Medicine, Department of Pediatrics Amalia Children's Hospital, Radboudumc Nijmegen The Netherlands

1

The human *BCS1L* gene encodes a homolog of *Saccharomyces cerevisiae* bcs1 protein involved in the assembly of complex III of the mitochondrial respiratory chain. Phenotypes associated with pathogenic biallelic *BCS1L* variants range from growth retardation, aminoaciduria, cholestasis, iron overload, lactic acidosis and early death (GRACILE) syndrome; MIM#603388) to Bjornstad syndrome (MIM#262000), characterized by abnormal flattening and twisting of hair shafts (pili torti) and sensori‐neural hearing loss without intellectual impairment. No causal treatment for this mitochondrial disease is available.

Ketogenic diets stimulate mitochondrial biogenesis, improve mitochondrial function and decrease oxidative stress^1^, ^2^, ^3^, ^4^ and have been implemented in some patients with mitochondrial disease and epilepsy.^5^, ^6^, ^7^

**Figure 1:**
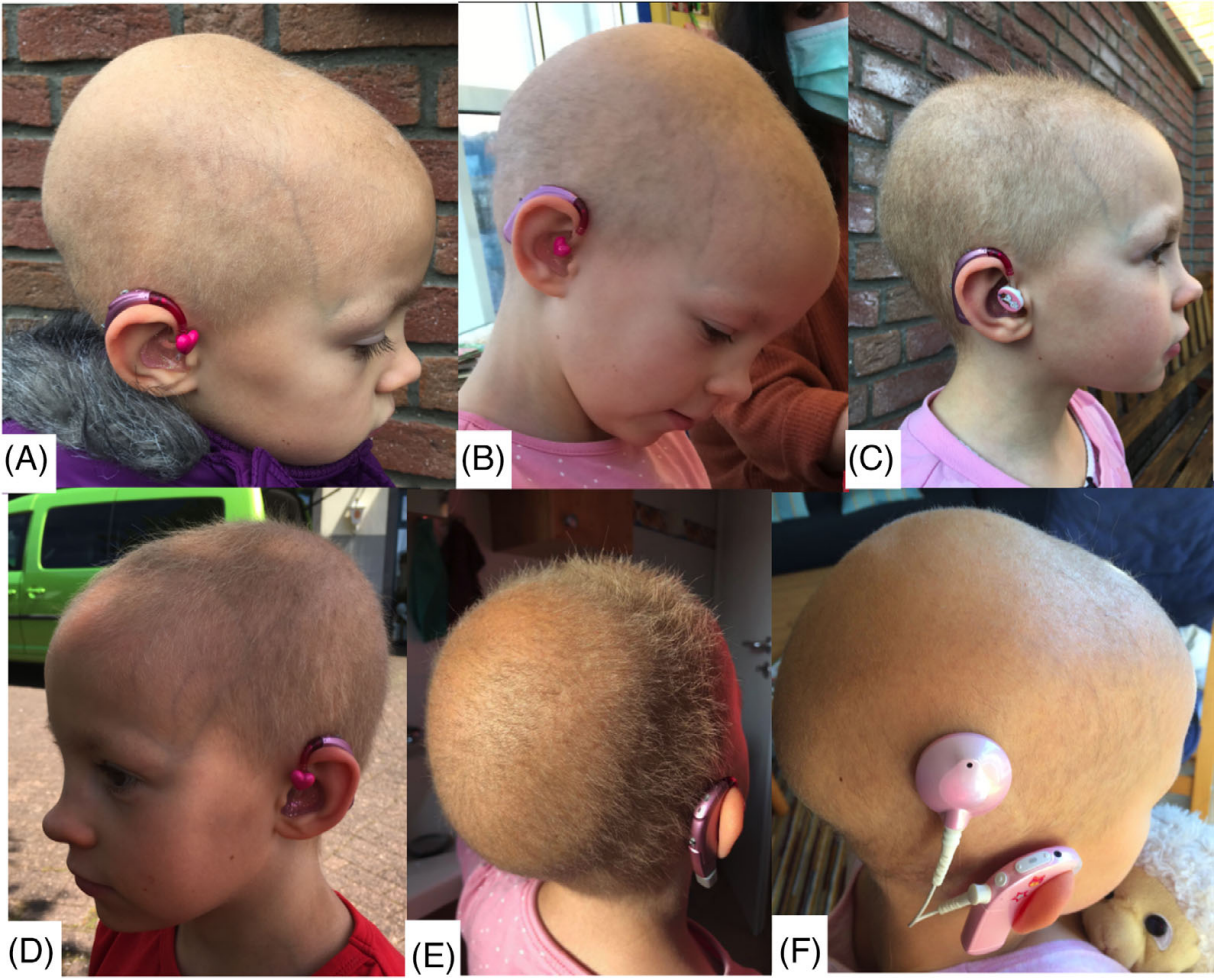
Patient with BCS1L related mitochondrial disease (Bjornstad syndrome) 2 months before (A), at day of starting (B) and after three (C) and four (D, E) months of modified Atkins diet. (F) Shows the patient 6 months after cessation of modified Atkins diet

We report on a 7‐year‐old female (*BCS1L*, NM_001079866.2, c.232A>G; p.Ser78Gly, c.794G>A; p.Arg265Gln) with very sparse and brittle hair since birth (Figure 1A,B), with progressive sensori‐neural hearing loss noted from age 18 months onwards. A 3‐day dietary history indicated an adequate diet with no protein deficiency but aminoaciduria, as a possible contributing cause for alopecia, was not confirmed or excluded. We initiated a mild ketogenic diet (modified Atkins diet [MAD] with 30‐40 g/day carbohydrates [1500 kcal/day], 10% of energy from carbohydrates, 25% from protein and 65% from fat). This was well tolerated and blood ketone levels of 2‐4 mmol/L were achieved.^8^ Hair growth improved during treatment (Figure 1C‐ E). Despite this, the family decided to go back to a regular diet after 4 months of diet because of the child craving for carbohydrates, and lack of improvement of hearing. Figure 1F shows that the hair was lost again 6 months after cessation of MAD.

There was improved hair growth during MAD in this individual with BCS1l related mitochondrial disease, We can only speculate if continuation of MAD would have further increased the hair growth or would have influenced the hearing loss and what effects an earlier initiation of MAD would have had.
